# Trends in male infertility burden in South Asia: a 30-year analysis of DALYs, prevalence, and future projections based on GBD 2021

**DOI:** 10.3389/frph.2025.1697925

**Published:** 2025-11-06

**Authors:** Kaleem Maqsood, Tahir Mehmood, Humayoun Huma Maqbool, Ali Afzal, Muhammad Faisal Maqbool, Mahnoor Fatima, Tayyba Jan, Samina Ashraf, Muhammad Babar Khawar, Chatchai Muanprasat

**Affiliations:** 1 School of Basic Medical Sciences & School of Public Health, Faculty of Medicine, Yangzhou University, Yangzhou, China; 2 Department of Biology, Lahore Garrison University, Lahore, Pakistan; 3 Chakri Naruebodindra Medical Institute, Faculty of Medicine Ramathibodi Hospital, Mahidol University, Bangpla, Bangplee, Thailand; 4 Institute of Zoology, University of the Punjab, Lahore, Pakistan; 5 Shenzhen Institutes of Advanced Technology, Chinese Academy of Sciences, Shenzhen, Guangdong, China; 6 University of Chinese Academy of Sciences, Beijing, China; 7 Centre of Excellence in Molecular Biology (CEMB), University of the Punjab, Lahore, Pakistan; 8 Department of Environmental Science, Lahore College for Women University, Lahore, Pakistan

**Keywords:** male infertility, disability-adjusted life years, prevalence, South Asia, Global Burden of Disease

## Abstract

**Purpose:**

Male infertility is an increasingly recognized global public health issue. Despite its impact, comprehensive assessments of long-term trends and regional disparities remain limited. Our study focuses on evaluating global and regional trends, particularly in South Asia, in disability-adjusted life years (DALYs) and prevalence of male infertility, and forecasting future patterns.

**Methods:**

Data from the Global Burden of Disease 2021 were analyzed for the period 1990–2021 to assess trends in DALYs and prevalence. The estimated annual percentage change (EAPC) was used to quantify changes over time. The Autoregression, Integrated, and Moving Average (ARIMA) model was applied to forecast future prevalence and DALYs trends in South Asia.

**Results:**

Globally, DALYs due to male infertility increased by 17.79% (EAPC 0.51%), and prevalence rose by 16.90% (EAPC 0.50%). South Asia showed a higher burden, with DALYs increasing by 45.66% (EAPC 1.40%) and prevalence by 47.19% (EAPC 1.50%). India showed the greatest rise, with DALYs and prevalence increasing by 55.87% and 58.82%, respectively. The most affected was the 25–29 years age group. A positive association was observed between male infertility burden and sociodemographic index (SDI) values across South Asia. Forecasts predict a continued rise in prevalence, while DALYs may slightly decline by 2030.

**Conclusion:**

Male infertility is increasing worldwide, with South Asia experiencing the most pronounced burden. While rising trends suggest improved detection, healthcare inequalities persist. Targeted interventions are essential to mitigate the growing impact of male infertility in the region.

## Introduction

Infertility is a major reproductive health issue worldwide, with a noteworthy psychological impact on the affected couples. Male infertility is defined as the failure to achieve conception after one year of unprotected intercourse. Literature suggests that male factors are solely responsible for an estimated 20%–30% occurrences of infertility (1), and male factors account for an estimated 20–30% of infertility cases (2). The evaluation of male infertility involves multiple parameters such as abnormal morphology of sperm, low sperm count, low volume production and ejaculation of sperm, reduced mobility of sperm, and increased time for sperm liquefaction (3).

The global burden of male infertility cases poses a major concern for public health, affecting nearly 56 million people throughout the world (4). Infertility represents a major psychological and social distress and additionally poses a financial challenge to people and healthcare systems (5). The decreasing fertility rates bring about social hurdles, particularly the aging population phenomenon. An investigation suggested that the global population is expected to hit its maximum number in 2064, whereas by 2100, it is estimated that 183 countries may have fertility rates below the replacement levels (6). This kind of population shift due to low fertility levels will have negative consequences for global development. Economic impacts can be shown in the increased healthcare expenditure and burden on the entire social security. Moreover, infertile men experience related depression and anxiety, and these conditions affect the well-being of individuals even more (7, 8). The impact of overall lifestyle, behavioral patterns, ethnicity, work stress, and associated factors upon health differs greatly between developed and developing countries (9). Thus, there is an urgent need to focus more on the activities that should help to stimulate fertility and combat the problem of infertility and reproductive health concerns.

Male infertility is caused by several different reasons. Most of these causes are not well understood (10). Male infertility-related diseases mainly include obesity (11) and high body mass index (BMI >29), as a higher BMI may result in a reduction in the total number of motile sperm cells. Similarly, the fragmented DNA tends to be higher in males with BMI >25 (12). Other contributing factors include hypogonadotropic hypogonadism, reproductive infections (13), and lifestyle factors (e.g., smoking, alcohol use, obesity, stress, and sedentary behavior), which have been reported to negatively regulate sperm quality and maturity. DNA damage appears to be highly affected by alcohol consumption than by smoking (14–16).

Many investigations point out that environmental endocrine disruptors have a key role in the occurrence of infertility. One such mechanism is testicular hypoplasia syndrome, which is caused by exposure to environmental endocrine disruptors (17, 18). Evidence suggests that semen quality may be influenced by environmental factors and lifestyle, which results in infertility (19).

Fertility issues affect millions of people worldwide, with a much higher incidence observed in developing countries (20). These countries confront major setbacks in the realms of social welfare, public health, and economic growth, making them crucial for addressing the wider effects of male infertility.

Given the widespread prevalence of male infertility, it is imperative to concentrate on specific regions to better understand its impact and create focused approaches. In comparison with female infertility, male infertility has not gotten as much attention, particularly in developing regions. This study presents the findings from the Global Burden of Disease (GBD) 2021 and assesses the current trends of disease burden of male infertility in South Asian countries from 1990 to 2021. Given the limited data available on male infertility in this region, this analysis is crucial for understanding the evolving public health impact and guiding future healthcare strategies.

## Methodology

### Data resources and definitions

This study utilized the GBD database to analyze the burden of male infertility. With data on 371 injuries and ailments as well as 88 significant risk factors for over 200 nations and territories, the GBD 2021 database is the broadest source of its kind. Additionally, it includes data from 1990 to 2021 (http://ghdx.healthdata.org/gbd-results-tool).

To generate global estimates of disease burden, particularly in those areas where such estimates are insufficient, a combination of various sources (including national health surveys, statistical modeling, and expert opinion) is used to generate the GBD estimates. The sampling methodology of the GBD database is strongly influenced by the availability of data in various countries or regions. That is, the mortality and morbidity data are presented as such for the nations with functional vital registration systems. In the absence of such data, there is a projection of disease burden by use of statistical modeling that combines household surveys, hospital data, and expert opinion.

Regarding inclusion and exclusion criteria, the International Classification of Diseases (ICD) coding system is applicable in categorizing various diseases, which may include male infertility. The ICD code of male infertility is GB04. This category also includes subcategories such as azoospermia (GB04.0), other specified male infertility (GB04.Y), and unspecified male infertility (GB04.Z). Coupled to these, male infertility caused by drugs leading to testicular hypoplasia falls under 5A81.1, while male infertility caused by cystic fibrosis falls under CA25.0, CA25.1, and CA25.Z. The GBD database compiles epidemiological estimates based on health records and reports provided by medical professionals rather than direct clinical diagnosis. Therefore, inclusion was limited to cases coded under the above ICD categories, while conditions outside these codes were excluded. Data were accessed on 10 July 2025, from the publicly available GBD database, which does not contain any identifiable participant information.

### Descriptive analysis

To explain the burden of male infertility more comprehensively in the countries of South Asia across 1990 and 2021, this study observed and acquired two measures: the age-standardized prevalence rate (ASPR) and age-standardized DALY rate (ASDR). The direct standardization method was used to calculate the age-standardized rates (ASR) by assigning the country-specific prevalence rates to a standard population to compensate for country variation in age distribution.

For age standardization, the World Standard Population as defined by the World Health Organization (WHO) was employed to calculate the rates. This method guarantees that variations in age distributions of different countries will not skew the comparisons between them. Standardization ages followed the following groups: 15–19, 20–24, 25–29, 30–34, 35–39, 40–44, and 45–49 years. Sociodemographic index (SDI) is a standardized composite measure that can be compared across countries and over time and has a scale of 0–1. It is computed through per capita income, the average number of years of schooling of people above the age of 15 years, and the total fertility rate (21).

### Statistical analyses

The estimated annual percentage change (EAPC), disability-adjusted life years (DALYs), prevalence, and their age-standardized rates are the key metrics used to evaluate the male infertility burden from 1990 to 2021 in South Asia. Maps are generated to illustrate the geographic distribution of rates in the years 1990 and 2021. Age-standardized rate trends over a stipulated time are measured by EAPC. The results were calculated by using the regression equation *y* = *α* + *βx* + *ε*, where *x* stands for the current year and *y* is the natural logarithm of the rate. The EAPC's 95% confidence interval (CI) was estimated by using the regression model, and the EAPC value was established as 100 [exp (*β*) − 1]. Negative lower limit of its 95% CI and EAPC value suggested a falling rate, whereas positive upper limit of its 95% CI and EAPC value suggested a growing rate.

The relationship between the burden of male infertility and SDI values was measured as the locally weighted scatterplot smoothing (LOWESS) method. The SDI and disease rates were of expected values integrated per country in South Asia. The degree, number, and position of knots were chosen automatically, and the parameter codifying the degree of smoothness of the fit was the span.

### Forecasting

**Figure 1:**
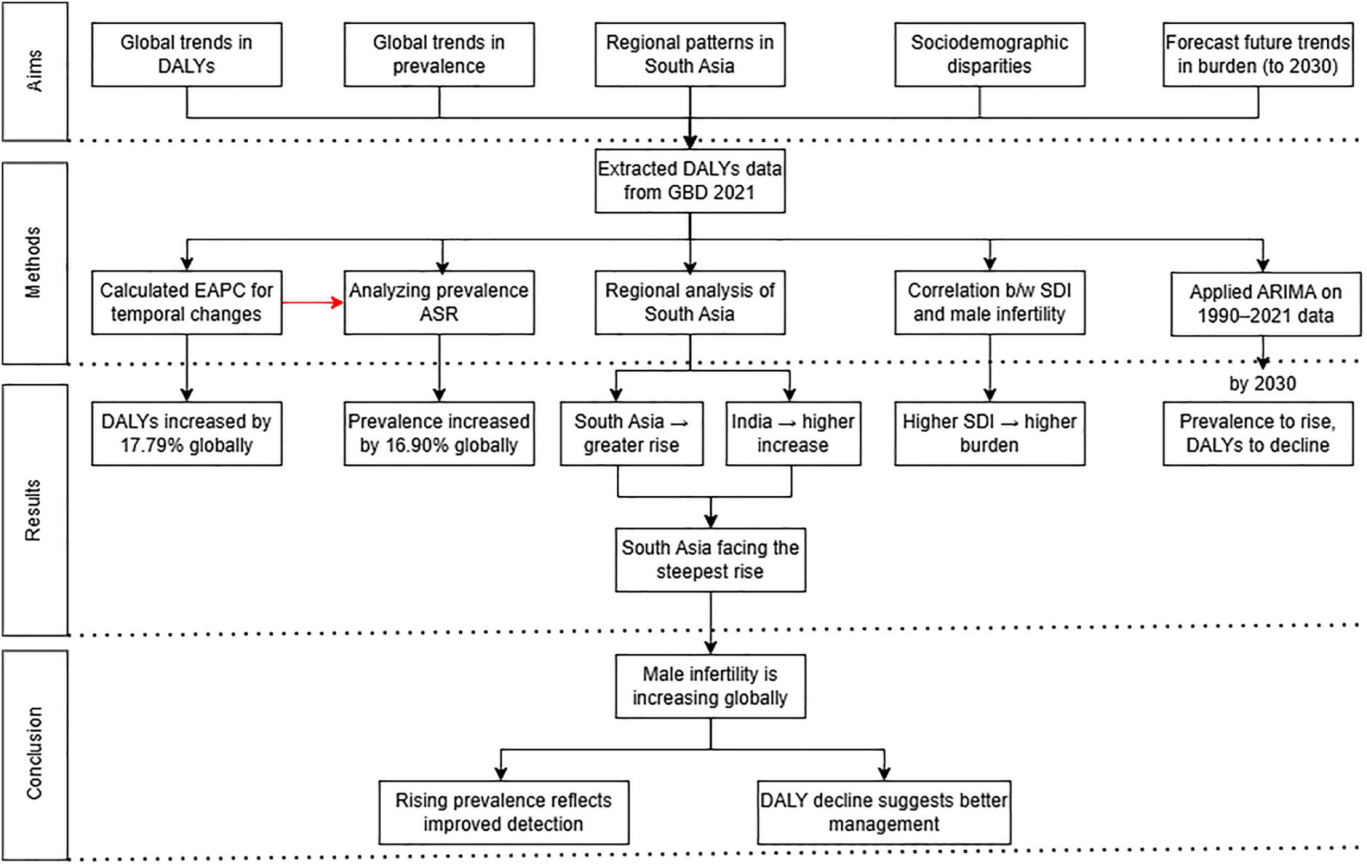
Flowchart for analysis of male infertility burden in South Asia.

In this study, we applied the ARIMA model to forecast the ASDR and prevalence of male infertility in South Asia. The first step involved ensuring that the data were stationary, as required for ARIMA modeling. We performed a Shapiro–Wilk test for normality, which revealed non-normal distributions for both the DALYs and prevalence datasets, prompting the use of differencing to achieve stationarity. The augmented Dickey–Fuller (ADF) test confirmed that the differenced data were stationary, making them suitable for ARIMA modeling. The model was selected by analyzing the autocorrelation function (ACF) and partial autocorrelation function (PACF) plots, which guided the choice of model parameters. We used the Akaike Information Criterion (AIC) for DALYs and the Bayesian Information Criterion (BIC) for prevalence to select the optimal model. The final models, ARIMA (2,2,1) for DALYs and ARIMA (2,2,0) for prevalence, were validated through residual diagnostics, including the Ljung–Box test, which confirmed the adequacy of the models. Forecasts were generated for the next decade, and confidence intervals were calculated to reflect uncertainty in the long-term projections. Data were analyzed and displayed using R statistical software (version 4.4.3). A summary map for the analysis is shown in Figure 1.

## Results

### DALY trend

The global ASDR for male infertility has shown an overall increase from 6.65 (95% CI: 2.47, 15.61) in 1990 to 7.84 (95% CI: 2.85, 18.56) in 2021. Despite this, the trend exhibits a moderate positive change, with an EAPC of 0.51% over the 1990–2021 period. The total DALY count in 2021 was 317,614, reflecting a growth of 17.79% in DALY rates during this period, as given in Table 1.

**Table 1:** ASR per 100,000 of DALYs, numbers, and percentage change for 1990 and 2021 with the estimated annual percentage change (EAPC) from 1990 to 2021.

Location	ASR per 100,000, 1990 (95% CI)	ASR per 100,000, 2021 (95% CI)	Numbers, 2021	PC 1990–2021	EAPC, %, 1990–2021
Global	6.65 (2.47,15.61)	7.84 (2.85,18.56)	317,614	17.79 (9.77,26.43)	0.51 (0.37,0.65)
Asia	6.89 (2.49,16.43)	8.36 (3.02,19.63)	207,795	21.22 (8.39,35.52)	0.61 (0.42,0.80)
South Asia—WB	5.88 (2.12,13.78)	8.56 (3.08,19.87)	91,289	45.66 (25.08,69.67)	1.40 (0.86,1.95)
Afghanistan	4.36 (1.48,10.77)	3.31 (1.34,7.32)	475	−24.01 (−48.04,20.33)	−1.23 (−1.48,−0.99)
Bangladesh	3.65 (1.45,7.79)	4.44 (1.78,9.44)	3,835	21.58 (5.16,38.34)	0.79 (0.49,1.08)
Bhutan	4.31 (1.47,10.35)	4.19 (1.45,9.87)	20	−2.84 (−11.24,5.91)	−0.09 (−0.10,−0.07)
India	5.71 (2.04,13.01)	8.90 (3.22,20.61)	72,582	55.87 (28.62,91.15)	1.91 (1.43,2.39)
Maldives	10.79 (3.92,26.41)	8.96 (3.59,19.27)	49	−16.95 (−42.68,22.70)	−0.95 (−1.41,−0.50)
Nepal	4.99 (1.72,11.89)	2.94 (1.16,6.15)	460	−41.14 (−58.65,−11.46)	−0.79 (−1.43,−0.14)
Pakistan	10.27 (2.89,26.93)	10.76 (2.74,31.09)	13,416	4.71 (−18.66,36.72)	−3.30 (−6.32,−0.19)
Sri Lanka	2.99 (1.19,6.55)	4.21 (1.52,9.85)	453	40.69 (−7.97,109.93)	0.42 (0.17,0.68)

South Asia – WB stands for World Bank-defined South Asia region.

Asia experienced a steady increase in ASDR, rising from 6.89 (95% CI: 2.49, 16.43) in 1990 to 8.36 (95% CI: 3.02, 19.63) in 2021, with a 21.22% increase over the study period. The EAPC stood at 0.61%, indicating a modest rise in the region. In 2021, the total DALY count for Asia was 207,795 as given in Table 1.

However, South Asia experienced a substantial increase in DALYs, with the region's DALY count rising by 45.66%. The EAPC for South Asia stood at 1.40%, suggesting a relatively higher increase compared with other regions. The DALY count in South Asia for 2021 was 91,289 as mentioned in Table 1.

### Country-wise analysis

**Figure 2:**
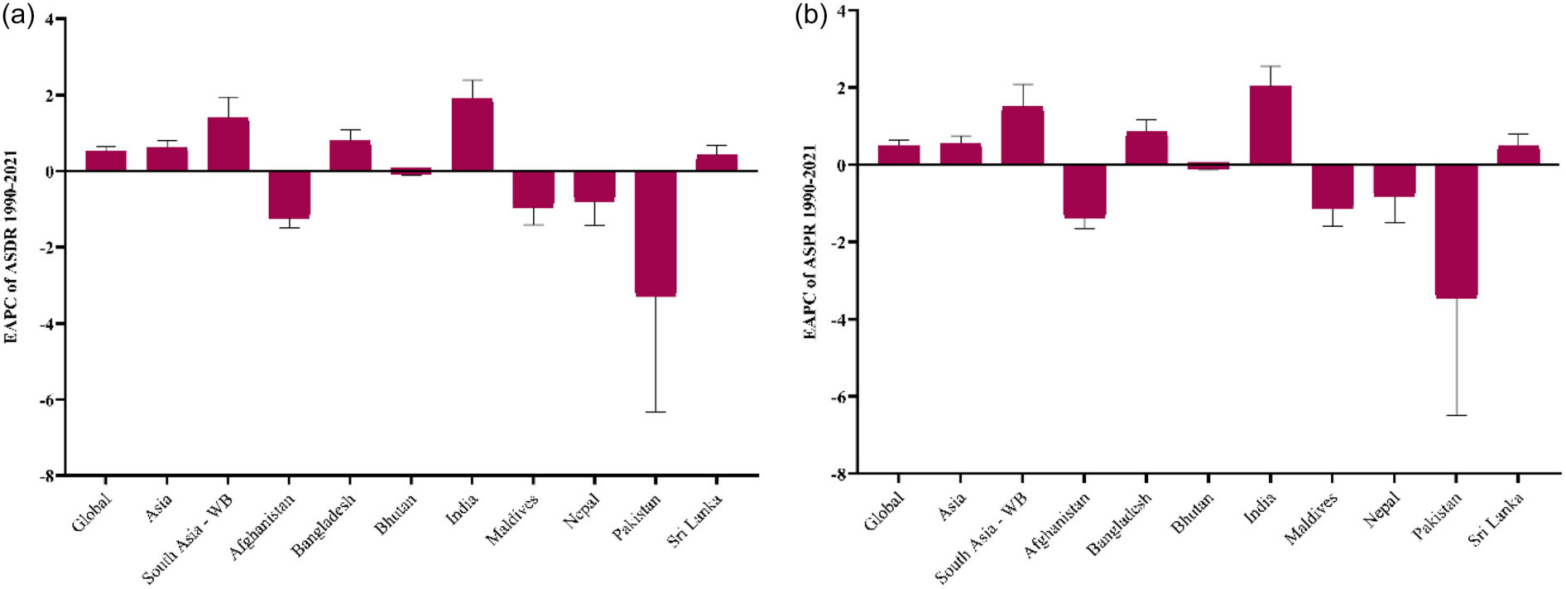
Estimated annual percentage change (EAPC) of **(a)** DALYs and **(b)** prevalence of male infertility from 1990 to 2021. Positive EAPC values indicate an increasing trend, while negative values indicate a decreasing trend. Country-level variations highlight differences in the temporal burden across the region.

**Figure 3:**
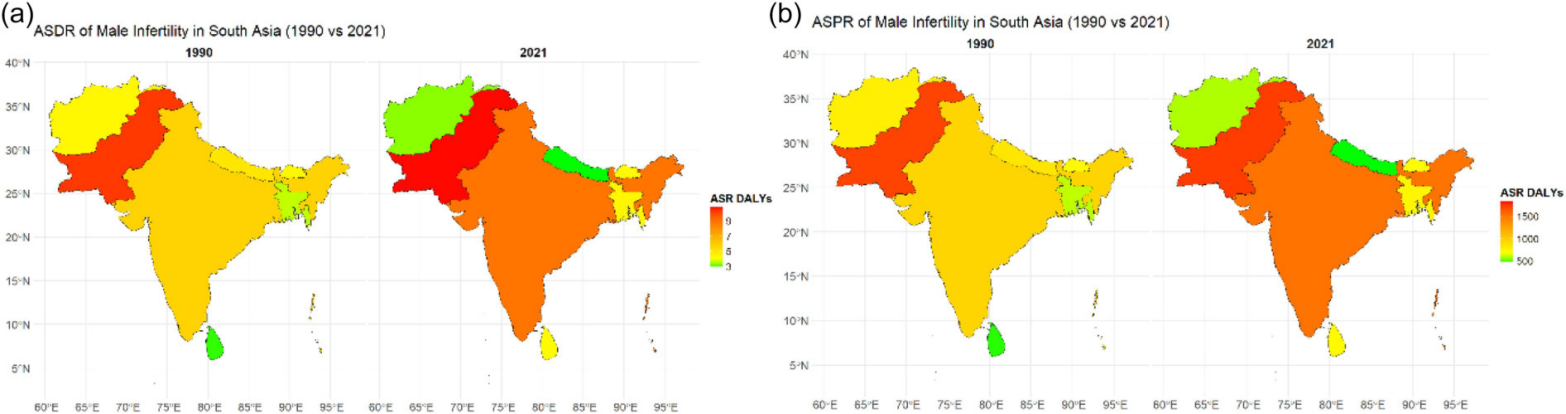
Choropleth map showing ASR per 100,000 of **(a)** DALYs and **(b)** prevalence for male infertility in South Asia: a comparison between 1990 and 2021. The darker shades indicate higher rates. Countries with higher ASR in 2021 compared with 1990 reflect an increasing burden, while the lighter shades indicate a decline.

India showed the most significant increase in DALYs related to male infertility, rising by 55.87% with an EAPC of 1.91%, the highest EAPC by a country as presented in Figure 2a, reflecting a sharp upward trend. Pakistan experienced a more moderate increase of 4.71%, with a slight decline earlier in the study period, but the highest ASDR among South Asian countries as presented in Figure 3a. Bangladesh saw a 21.58% increase in DALYs, with an EAPC of 0.79%, and Sri Lanka showed a 40.69% rise with an EAPC of 0.42%. In contrast, Nepal and Afghanistan both experienced declines, with Nepal seeing a significant reduction of 41.14% and Afghanistan a decrease of 24.01%, with both showing negative EAPC. The Maldives also showed a 16.95% decrease in DALYs, reflecting a decline in the health burden. Bhutan experienced a slight decline of 2.84% in DALYs, accompanied by a slightly negative EAPC, as shown in Table 1.

### Age groups trend in South Asia (2021)

**Figure 4:**
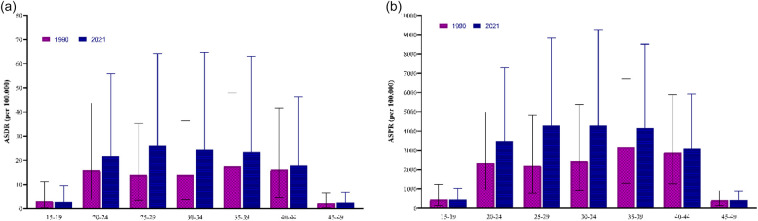
Disability-adjusted life years and prevalence of male infertility by age group in South Asia. Age-specific **(a)** DALYs and **(b)** prevalence rates of male infertility in South Asia for the years 1990 and 2021.

**Figure 5:**
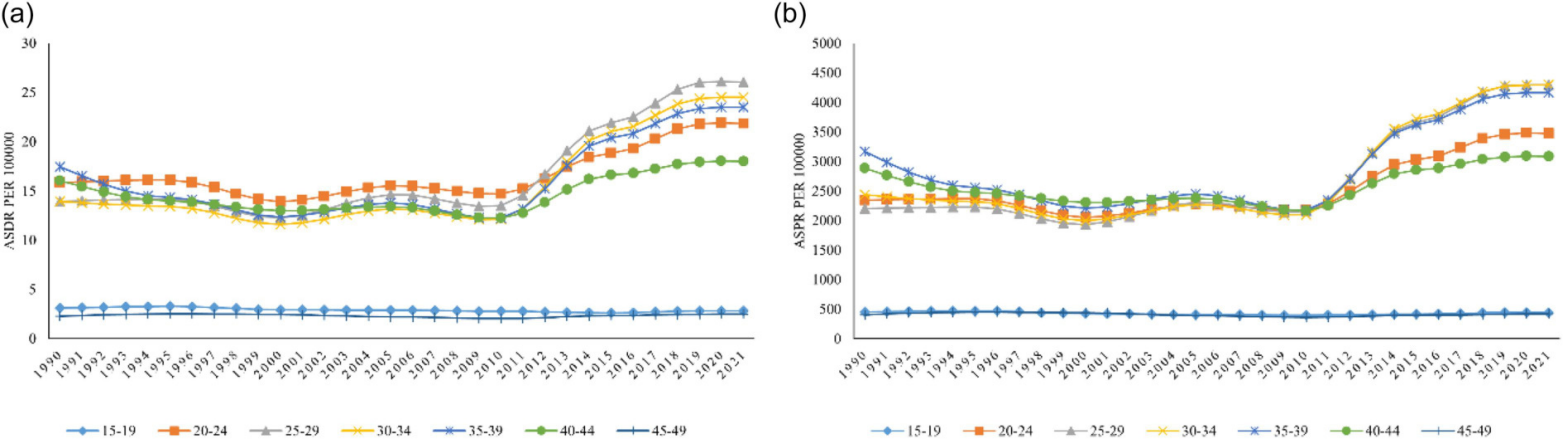
Trends in DALYs and prevalence rate during 1990–2021. Age-specific trends in **(a)** DALYs and **(b)** prevalence rate of male infertility in South Asia from 1990 to 2021. Data are presented for multiple age groups, highlighting temporal changes and differences across age categories. Values are based on estimates from the Global Burden of Disease 2021 study.

The DALYs for male infertility in South Asia show a similar trend, with the highest rates in the 25–29 years age group (26.05), reflecting a substantial burden of male infertility in late adolescence and early adulthood. DALYs remain relatively consistent in the 30–39 years group, indicating an ongoing impact during the thirties. However, DALYs decline sharply in the 40–44 years group (18.02) and more significantly in the 45–49 years group (2.48), suggesting a reduction in the health burden of male infertility as individuals age (Figure 4a). Over the study period of 1990–2021, a growing trend of DALYs was observed in the 25–29 years age group (Figure 5a). Component analysis of DALYs indicated that years of life lost (YLL) were consistently zero across all years, reflecting the non-fatal nature of male infertility. Thus, DALYs were entirely driven by years lived with disability (YLD) (Supplementary Table S1).

### Prevalence trend

Table 2 shows the global and country-wise trend of the prevalence of male infertility. The global trend of prevalence of male infertility has steadily increased from 1,158.86 (95% CI: 696.62, 1,858.35) in 1990 to 1,354.76 (95% CI: 802.12, 2,174.77) in 2021, showing a 16.90% increase in prevalence. The EAPC for the global prevalence was 0.50%, indicating a modest upward trend. In 2021, the total prevalence count globally was 55,000,818.

**Table 2:** ASR per 100,000 of prevalence, numbers, and percentage change for 1990 and 2021 with the estimated annual percentage change (EAPC) from 1990 to 2021.

Location	ASR per 100,000, 1990 (95% CI)	ASR per 100,000, 2021 (95% CI)	Numbers, 2021	PC 1990–2021	EAPC, %, 1990–2021
Global	1,158.86 (696.62,1,858.35)	1,354.76 (802.12,2,174.77)	55,000,818	16.90 (9.19,24.91)	0.50 (0.36,0.64)
Asia	1,223.56 (713.10,1,990.76)	1,451.00 (854.12,2,335.70)	36,186,658	18.59 (6.34,32.12)	0.56 (0.37,0.75)
South Asia—WB	982.05 (562.83,1,590.51)	1,445.48 (827.44,2,369.28)	15,391,726	47.19 (24.72,74.31)	1.50 (0.92,2.08)
Afghanistan	793.73 (432.47,1,317.15)	585.09 (432.08,764.44)	82,555	−26.29 (−50.32,13.53)	−1.39 (−1.66,−1.12)
Bangladesh	604.00 (449.12,797.59)	741.06 (554.73,981.89)	640,268	22.69 (7.33,38.42)	0.85 (0.52,1.17)
Bhutan	725.10 (423.27,1,183.84)	699.68 (409.08,1,141.15)	3,411	−3.51 (−10.56,2.59)	−0.10 (−0.12,−0.09)
India	954.95 (542.40,1,579.69)	1,516.62 (872.42,2,491.07)	12,352,775	58.82 (28.90,97.85)	2.03 (1.52,2.55)
Maldives	1,837.26 (1,068.04,2,979.91)	1,459.44 (1,109.85,1,890.73)	8,044	−20.56 (−46.52,17.65)	−1.14 (−1.59,−0.68)
Nepal	838.86 (499.05,1,382.62)	475.37 (350.54,629.31)	73,946	−43.33 (−60.95,−15.70)	−0.83 (−1.51,−0.15)
Pakistan	1,718.87 (655.31,3,406.67)	1,735.86 (602.15,3,629.48)	2,152,785	0.99(−19.77,30.50)	−3.46 (−6.49,−0.33)
Sri Lanka	481.90 (355.55,644.63)	722.34 (406.77,1,204.21)	77,942	49.89 (−1.55,132.05)	0.50 (0.20,0.80)

The prevalence rate in Asia increased by 18.59% over the study period, with a slight increase in EAPC at 0.56%, reflecting an overall upward trend in male infertility cases. The prevalence count for Asia in 2021 was 36,186,658. South Asia experienced a notable rise in prevalence, with a 47.19% increase. The EAPC of 1.50% indicates a higher rate of increase compared with other regions. In 2021, the prevalence count for South Asia was 15,391,726.

### Country-wise analysis

India experienced the most significant rise in prevalence of male infertility, with a 58.82% increase and the highest EAPC of 2.03% among South Asian countries as presented in Figure 2b, indicating an alarming upward trend. In contrast, Pakistan saw a minimal increase of 0.99%, although it experienced some fluctuation over the years, with a negative EAPC of −3.46%, suggesting a slight overall decline, but the highest ASPR among South Asian countries as presented in Figure 3a. Bangladesh showed a moderate increase in prevalence, rising by 22.69% with an EAPC of 0.85%, indicating a gradual rise in cases. Nepal and Afghanistan both experienced significant declines, with Nepal seeing a 43.33% reduction and a negative EAPC of −0.83% and Afghanistan exhibiting a 26.29% decrease with an EAPC of −1.39%. The Maldives also showed a decline of 20.56% in prevalence, with a negative EAPC of −1.14%, while Bhutan had a smaller decline of 3.51%, with a slightly negative EAPC of −0.10%. Sri Lanka showed a moderate increase of 49.89% in prevalence, with an EAPC of 0.50%, reflecting a gradual rise in male infertility cases over the period.

### Trend by age group in South Asia (2021)

The prevalence of male infertility in South Asia in 2021 shows significant variation across age groups. The highest prevalence was found in the 20–24 years age group (3,472.37), followed closely by the 25–29 years age group (4,295.67), indicating a peak in male infertility during early adulthood. Prevalence then gradually declined in the older age groups, with the lowest rate observed in the 45–49 years age group (419.05). The data highlight that male infertility is most prevalent in younger adult males, especially between 20 and 29 years, before decreasing in older age groups, as presented in Figure 4b. Over the study period of 1990–2021, a growing trend of prevalence was observed in the 25–29 years age group (Figure 5b).

### Relationship between male infertility burden and SDI

**Figure 6:**
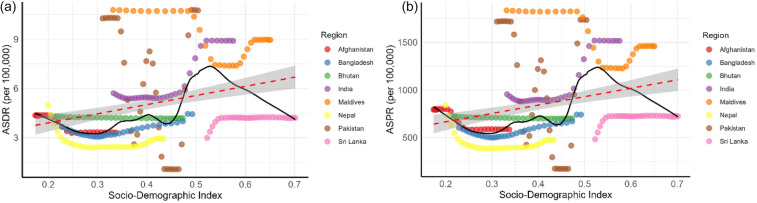
Association between age-standardized **(a)** DALYs and **(b)** prevalence rates of male infertility and sociodemographic index from 1990 to 2021, in South Asian countries. The black curve represents the smoothed fitting curve based on the LOWESS method, the red curve represents the linear regression curve, and the shaded area represents the 95% confidence interval.

The relationship between age-standardized DALY rates, prevalence rates of male infertility, and SDI values in South Asian countries from 1990 to 2021 is shown in Figure 6.

Figure 6a demonstrates that as SDI increases, DALY rates for male infertility rise, particularly in countries such as India and Sri Lanka, indicating a stronger recognition of male infertility due to better healthcare access. The LOWESS smoothed curve (black) captures this upward trend, while the linear regression line (red) also shows a positive association between SDI and DALY rates.

Figure 6b shows a similar trend for prevalence rates of male infertility, with higher SDI correlating with higher prevalence, supporting the idea that increased awareness and healthcare access lead to more diagnoses. Both panels highlight a positive association between SDI and the burden of male infertility, with confidence intervals showing the variability in the trends.

### Forecast to 2030 in South Asia

**Figure 7:**
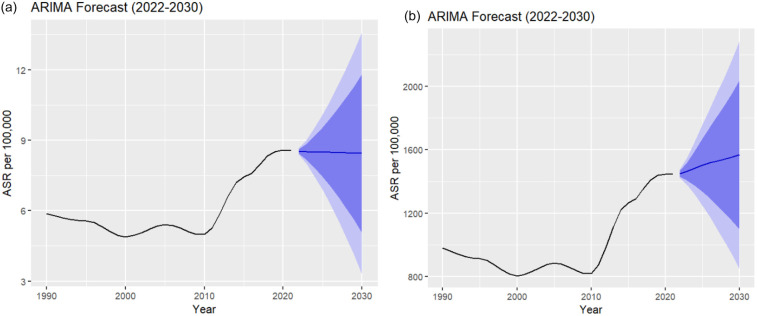
ARIMA model forecasts of ASR per 100,000 male infertility in South Asia by **(a)** DALYs and **(b)** prevalence from 2022 to 2030.

The ARIMA model was applied to analyze the ASDR and ASPR of male infertility in South Asia. For DALYs [ARIMA (2,2,1)], the forecast for the next 9 years (2022–2030) shows a gradual decline in the ASR, reflecting improvements in mortality rates over time. The forecasted values in Table 3 show widening confidence intervals, indicating increasing uncertainty as the forecast horizon extends. However, for prevalence [ARIMA (2,2,0)], the forecast indicates a gradual increase in the ASR for male infertility in South Asia over the same period, with the values reflecting a steady upward trend in the projected data. Figure 7 shows the ARIMA model forecasts of ASR per 100,000 male infertility in South Asia by (a) DALYs and (b) prevalence from 2022 to 2030.

**Table 3:** Forecasted male infertility measures: DALYs and prevalence ARIMA for 9 years forecast with 95% confidence interval.

Year	DALYs			Prevalence		
	Forecast	Lower 95	Upper 95	Forecast	Lower 95	Upper 95
2022	8.5254	8.378	8.673	1,449.657	1,420.5849	1,478.729
2023	8.5053	7.987	9.024	1,464.288	1,375.2423	1,553.334
2024	8.4984	7.467	9.529	1,484.910	1,315.3822	1,654.438
2025	8.4934	6.887	10.100	1,504.313	1,247.8446	1,760.782
2026	8.4844	6.266	10.702	1,518.856	1,176.4629	1,861.249
2027	8.4729	5.599	11.346	1,529.815	1,102.0769	1,957.553
2028	8.4614	4.879	12.044	1,540.625	1,023.7141	2,057.537
2029	8.4511	4.106	12.796	1,553.557	939.7312	2,167.383
2030	8.4412	3.284	13.598	1,568.481	849.0056	2,287.957

## Discussion

We have performed a comprehensive analysis of the global and regional trends in DALYs and prevalence rates of male infertility over the past three decades to understand the evolving health burden, particularly in South Asia. Our findings reveal a concerning upward trajectory in both prevalence and DALYs globally, with a significant rise in South Asia. Globally, the prevalence of male infertility and the DALYs associated with male infertility increased. This combination suggests that as more men are being diagnosed with infertility, the impact on their quality of life is becoming more significant. Levine et al. (22) published an article showing that sperm concentrations were declining in the USA and around the world (22). The observed rise in male infertility cases is attributed to declining semen quality worldwide (3). A recent study by Shan et al. (23) reported a similar trend in China that the DALYs and prevalence of male infertility are on the rise in a country that accounts for one-fifth of the global reported male infertility cases. Research implies that male infertility could be an indication of poor general health in the long run. Not only is there the possibility of infertility being a presentation symptom of an underlying disease, but men with deranged semen parameters are also more prone to malignancy (24).

In Asia, the increase in DALYs was significant, with South Asia experiencing a sharper rise. The EAPC for South Asia further reflects a more substantial increase compared with the global average. The rise in both DALYs and prevalence in South Asia is indicative of a region where male infertility is becoming a more significant public health concern. Another GBD study showed that South Asian men exhibited a significant increase in both primary and secondary infertility prevalence in recent years (25). This trend is likely driven by a combination of factors, including changing lifestyles, environmental factors, improved healthcare access, and more accurate reporting and diagnosis (26). Over the past few years, do-it-yourself sperm analysis tests have been widely commercialized to permit males to undertake a preliminary examination of their fertility. The vast majority of products show only binary (yes/no) results in sperm concentration based on the WHO-recommended cutoff values, either 15 or 20 M/mL (27). These indicate inexpensive and readily available means to diagnose a person who might have otherwise chosen not to seek medical attention. Studies in Poland have pointed out the fact that the reason behind such a high prevalence of infertility in the eastern part of Europe can also be related to sociopsychological reasons, which include a high rate of unemployment, excessive rate of daily stress, and late childbearing. Since these parameters are also on the rise in South Asia, the increasing rates of male infertility may be attributed to these (28).

India has shown the most dramatic increase in both DALYs and prevalence, highlighting a concerning trend in male infertility. Xu et al. (29) also concluded that BRICS with the highest growth in male infertility is India, followed by 8.51 million. Poor regulation of epidemiology, combined with unregulated population growth, has been a deciding factor. This sharp rise suggests that India is facing significant public health challenges in addressing male infertility, with potential factors including urbanization, dietary changes, pollution, and lifestyle factors such as stress and sedentary behavior. In India, some of the reasons attributable to low fertility parameters include high-level pollution and endocrine disruptors, which are common in the surroundings, have been shown to impair sperm quality and reproductive health (30). It is believed that in India, infertility is as prevalent as 45% because of local traditional culture and beliefs toward infertility, and most Indians believe that infertility is a curse from God and not the fault of men, who are not to be blamed for the issue of inability to conceive (31). In contrast, Pakistan's relatively modest increase in DALYs and prevalence may reflect limited healthcare access and awareness, pointing to potential underreporting or underdiagnosis of the condition. Another reason for underreporting is the stigmatization of male infertility in Pakistan, reflecting underdiagnosis and underreporting rather than a truly lower disease burden (32). Moreover, early marriage and early childbearing, common across South Asia, may increase the likelihood of infertility being detected at younger ages. The positive correlation observed between SDI and disease burden can be attributed to both improved healthcare access and lifestyle changes associated with higher SDI settings, such as sedentary behavior, dietary shifts toward high-sugar and high-fat intake, and increased stress, all of which may contribute to rising infertility. Similar findings have been reported previously, where increased prevalence of male infertility in South Asia was linked to better healthcare accessibility combined with lifestyle risks (25).

Other countries in South Asia, such as Bangladesh, Sri Lanka, and the Maldives, have experienced moderate increases in both DALYs and prevalence, which are indicative of growing recognition and diagnosis of male infertility. However, Nepal, Afghanistan, and Bhutan saw declines in DALYs and prevalence, which might suggest underreporting, limited healthcare access, or other factors that are affecting the accuracy of data collection in these regions. Shan et al. (23) attribute the innovations in various areas to include traditional Chinese medicine (TCM), development of assisted reproduction centers and sperm banks, emergence of microsurgery, and optimization of varicocele treatment, among others. Perhaps, these advances may explain the rising rates in the instance of the developing countries such as India, Bangladesh, and Sri Lanka, among others, where as yet these areas have failed to record the breakthrough. Afghanistan, on the other hand, may show a decline in cases because of stigmatization and unreported cases. The war in Afghanistan during the period of study may also have impacted the reporting of such cases.

The trends for male infertility in South Asia show the highest burden in the 25–29 years age group, both in terms of DALYs and prevalence. This suggests that male infertility is most impactful during the prime reproductive years. Another GBD study indicated a substantial increase in the prevalence of male infertility since 1990, and the 30–34 years age group showed the highest prevalence and YLD on a global scale (4). High prevalence and DALY rates in this age group underline the significant socioeconomic and psychological consequences for individuals affected by infertility at this stage in life. Chinese research established that in young adults, sperm density decreased at a more rapid pace in students compared with non-students. These findings indicate that a sedentary lifestyle, sleep disturbance, high psychosocial stress, and the lifestyle of college students who use smartphones and the Internet excessively negatively impact semen quality (33). The other known interesting factor causing male infertility is unintended side effects of various drugs in young men, which may reduce fertility and cause changes in the hypogonadal–pituitary–gonadal axis (34). Such drugs are chemotherapeutic agents, psychotropic drugs, long-term corticosteroid usage, male pattern baldness drugs, or testosterone replacement therapy. The drugs can disturb the parameters of semen or provoke sexual complications (35). The decline in DALYs and prevalence rates in older age groups (40–44 and 45–49 years) may reflect reduced treatment-seeking behavior, lower diagnosis rates, or possibly the natural reduction in fertility as men age. Another study by Shan et al. (23) found that in China, the most susceptible men are those aged 20–44 years, although the greatest prevalence and DALYs have been recorded in the 35–49 years age group since 1990.

The ARIMA model forecast indicates a steady increase in the prevalence of male infertility in South Asia, signaling that the condition's burden will continue to rise. While DALYs are expected to show a slight decline, the overall prevalence is projected to keep increasing, suggesting that more men will be diagnosed with infertility. The widening confidence intervals in the forecast emphasize the uncertainty in predicting long-term trends, yet they highlight the need for continued efforts to address the growing problem. Interestingly, our forecast revealed a continuous increase in the prevalence, while DALYs showed a slight decline. Component analysis indicated that YLL were consistently zero, reaffirming the non-fatal nature of this condition. Hence, DALYs were entirely driven by YLD. The observed decline in DALYs despite rising prevalence may reflect improvements in healthcare accessibility, early diagnosis, and better management strategies that reduce the severity and disability impact of infertility. Additionally, the growing availability of assisted reproductive technologies and home-based diagnostic kits may have expanded case detection, particularly of milder cases, thereby contributing to rising prevalence but reduced disability weight at the population level (27).

The rising global burden of male infertility, as indicated by both DALYs and prevalence trends, highlights significant public health concerns, particularly in South Asia. The data show a steady increase in the health impact of male infertility, with South Asia experiencing the most considerable rise. However, there are limitations in data accuracy and availability, as many regions, particularly those in South Asia, may face challenges related to underreporting due to cultural stigma and limited healthcare access. Estimates for some South Asian countries (e.g., Afghanistan and Nepal) rely on modeled data due to limited vital registration, which may bias intra-regional comparisons. Additionally, the factors contributing to male infertility, such as environmental influences, lifestyle choices, and genetic predispositions, remain inadequately explored, further limiting the understanding of the issue. Another limitation is that the GBD model projects trends only from historical data and cannot account for future interventions, environmental changes, or epidemics, leading to uncertainty in long-term predictions. Lastly, the use of the WHO World Standard Population may underestimate the burden in younger groups, as South Asia has a higher proportion of men aged 25–29 years; region-specific standards could improve accuracy. To address these gaps, future efforts should focus on improving data collection systems, particularly in low-resource settings, and reducing the social stigma surrounding male infertility through public awareness campaigns. Strengthening healthcare infrastructure and promoting preventive measures such as lifestyle changes could help mitigate the rising burden of male infertility. As the forecast suggests an upward trend in prevalence, timely interventions and long-term strategies will be essential to manage this growing health challenge.

## Conclusion

Our study reveals a significant increase in male infertility globally, with South Asia experiencing the highest rise in both DALYs and prevalence. India shows the most substantial increase, while Pakistan's modest rise may reflect underreporting or limited healthcare access. The highest burden is in the 25–29 years age group, indicating major socioeconomic impacts. The forecast to 2030 suggests a continued rise in prevalence, highlighting the need for improved healthcare access, awareness, and targeted public health interventions to address this growing issue in South Asia.

## Data Availability

The data are accessible through the Global Burden of Disease Study 2021, available at https://ghdx.healthdata.org/gbd-2021.
