# Supplementary material for: Trends in male infertility burden in South Asia: a 30-year analysis of DALYs, prevalence, and future projections based on GBD 2021
Source: Front Reprod Health. 2025 Nov 6;7:1697925. doi: 10.3389/frph.2025.1697925 (PMC12631329; doi:10.3389/frph.2025.1697925)
Supplement: Supplementary file 1 [file Table1.docx]

**Table S1.** Age-standardized rates (per 100,000 males) of Years Lived with Disability (YLD), Years of Life Lost (YLL), and Disability-Adjusted Life Years (DALYs) due to male infertility in South Asia, 1990–2021.

| Year | DALYs | YLDs | YLLs |
| --- | --- | --- | --- |
| 1990 | 3.04(1.10,7.13) | 3.04(1.10,7.13) | 0 |
| 1991 | 2.99(1.07,7.01) | 2.99(1.07,7.01) | 0 |
| 1992 | 2.95(1.05,6.88) | 2.95(1.05,6.88) | 0 |
| 1993 | 2.91(1.04,6.72) | 2.91(1.04,6.72) | 0 |
| 1994 | 2.88(1.03,6.63) | 2.88(1.03,6.63) | 0 |
| 1995 | 2.87(1.02,6.57) | 2.87(1.02,6.57) | 0 |
| 1996 | 2.83(1.00,6.43) | 2.83(1.00,6.43) | 0 |
| 1997 | 2.74(0.95,6.22) | 2.74(0.95,6.22) | 0 |
| 1998 | 2.63(0.92,6.01) | 2.63(0.92,6.01) | 0 |
| 1999 | 2.54(0.90,5.82) | 2.54(0.90,5.82) | 0 |
| 2000 | 2.50(0.89,5.73) | 2.50(0.89,5.73) | 0 |
| 2001 | 2.53(0.90,5.82) | 2.53(0.90,5.82) | 0 |
| 2002 | 2.59(0.91,5.92) | 2.59(0.91,5.92) | 0 |
| 2003 | 2.66(0.94,6.08) | 2.66(0.94,6.08) | 0 |
| 2004 | 2.72(0.96,6.29) | 2.72(0.96,6.29) | 0 |
| 2005 | 2.76(0.97,6.43) | 2.76(0.97,6.43) | 0 |
| 2006 | 2.73(0.96,6.27) | 2.73(0.96,6.27) | 0 |
| 2007 | 2.67(0.93,6.15) | 2.67(0.93,6.15) | 0 |
| 2008 | 2.59(0.91,6.04) | 2.59(0.91,6.04) | 0 |
| 2009 | 2.54(0.91,5.96) | 2.54(0.91,5.96) | 0 |
| 2010 | 2.53(0.90,5.98) | 2.53(0.90,5.98) | 0 |
| 2011 | 2.68(0.95,6.35) | 2.68(0.95,6.35) | 0 |
| 2012 | 2.99(1.08,6.93) | 2.99(1.08,6.93) | 0 |
| 2013 | 3.35(1.21,7.74) | 3.35(1.21,7.74) | 0 |
| 2014 | 3.65(1.34,8.34) | 3.65(1.34,8.34) | 0 |
| 2015 | 3.77(1.39,8.64) | 3.77(1.39,8.64) | 0 |
| 2016 | 3.85(1.41,8.75) | 3.85(1.41,8.75) | 0 |
| 2017 | 4.04(1.48,9.19) | 4.04(1.48,9.19) | 0 |
| 2018 | 4.23(1.56,9.70) | 4.23(1.56,9.70) | 0 |
| 2019 | 4.33(1.57,10.08) | 4.33(1.57,10.08) | 0 |
| 2020 | 4.35(1.58,10.07) | 4.35(1.58,10.07) | 0 |
| 2021 | 4.34(1.56,10.08) | 4.34(1.56,10.08) | 0 |
